# Analysis of Radiopaque Gastrointestinal Foreign Bodies Expelled by Spontaneous Passage in Children: A 15-Year Single-Center Study

**DOI:** 10.3389/fped.2018.00172

**Published:** 2018-06-12

**Authors:** Hung-Yu Yeh, Hsun-Chin Chao, Shih-Yen Chen, Chien-Chang Chen, Ming-Wei Lai

**Affiliations:** Division of Gastroenterology, Department of Pediatrics, Chang Gung Children's Medical Center, Chang Gung Memorial Hospital, College of Medicine, Chang Gung University, Taoyuan, Taiwan

**Keywords:** foreign body (FB), gastrointestinal (GI), radiopaque, transit time, spontaneous passage, children

## Abstract

**Background:** Most ingested foreign bodies (FBs) pass spontaneously through the gastrointestinal (GI) tract, but only limited data on transit time are available. We evaluated the relationship of FB size and shape with transit time.

**Methods:** We retrospectively reviewed medical records collected over 15 years (January 2001 to December 2015) on pediatric patients with radiopaque FBs in the GI tract. We categorized the FBs as regularly (round or spherical) or irregularly shaped (ovoid, long, flake-like, or projecting) and measured their sizes radiographically. The diameter of regularly shaped FBs and the length of irregularly shaped FBs were correlated with transit time.

**Results:** In total, 484 patients with GI FBs were surveyed, and 267 (55.1%) FBs were radiopaque. Among the 267 radiopaque FBs, 88 (33.1%) required endoscopic removal and 7 (2.6%) underwent surgical intervention. Eighty-seven patients with single FBs in the GI tract for whom precise details of transit time were enrolled into the analysis of transit time; their mean age was 3.48 ± 2.21 years. Of the 87 FBs, 61 (70.1%) were regularly shaped, and 26 (29.9%) were irregularly shaped. The diameter of regularly shaped FBs was positively associated with transit time, as revealed by Mann-Whitney *U* test; diameters >1.5 and >2 cm were significantly correlated with longer transit times (both *p* = 0.003). A trend toward an increased transit time for long irregularly shaped FBs was also apparent; the *p*-values for lengths of 1.5, 2, and 2.5 cm were 0.824, 0.153, and 0.055, respectively. Under receiver operating characteristic (ROC) curve analysis, the optimal cutoff diameter for regularly shaped FBs, and length for irregularly shaped FBs, to predict a transit time of longer than 72 h were 1.95 and 2.25 cm, respectively.

**Conclusions:** The passage rate of ingested radiopaque FBs is 64.4%. Small FBs that have passed the duodenal curve should be managed conservatively via clinical observation and radiographic surveillance. Our results indicate that the larger an FB is, the longer the transit time will be.

## Introduction

Most foreign body (FB) ingestions occur in children between the ages of 6 months and 3 years (1, 2). Children constitute up to 80% of patients that ingest FBs (3). Approximately 80–90% of FBs that reach the gastrointestinal (GI) tract pass spontaneously; 10–20% are removed endoscopically, and 1% by surgery (4–8). Referral for endoscopic removal is indicated if the FB is located in the esophagus, if multiple magnets have been swallowed, if the FB is sharp or large, and if symptoms/signs of bowel obstruction are present (1, 9).

As most FBs pass spontaneously, surgical removal is considered only if no radiographic progression is evident by 3 days after ingestion or if the patient becomes symptomatic (10). Therefore, observation with regular follow-up radiography is the norm. Most radiopaque FBs are metal objects such as coins, pins, screws, magnets, button-like batteries, and nails (11, 12). Fish bones, plastics, and food items are not radiopaque, except for some large or thick fish bones (13).

The transit time for an asymptomatic FB can be hours to weeks. Only a few reports have evaluated transit time. The guidelines contain little information on the impact of the size of a FB on the transit time (9, 13, 14). Herein, we assessed the transit time of spontaneous GI passage in pediatric patients and further evaluated the relationship between FB size and transit time.

## Methods

Over 15 years (January 2001 to December 2015), all pediatric patients (<18 years of age) who were diagnosed with GI tract FBs in the Emergency Department of Linkou Chang Gung Memorial Hospital were retrospectively evaluated by reviewing the medical records. The recruited patients were classified into endoscopic removal, conservative treatment, and surgery groups. We recorded gender, age at diagnosis, the diameter of regularly shaped FBs, and the length of irregularly shaped FBs, noted relevant history, and performed radiography. Our goal was to assess transit time and its relationship to the size of the FB. Parents or caregivers were told to watch for passage of the object in the stool. As radiopaque FBs are evident on plain film and can be measured, subjects with radiopaque FBs were enrolled. The FB size was correlated with the transit time.

### Exclusion criteria

We excluded patients treated in the outpatient department because we lacked data on precise transit times. Patients who had undergone abdominal surgery or who had pre-existing abnormalities of the GI tract (congenital megacolon, Meckel diverticulum, or strictures), active GI diseases (dyspepsia, constipation, or irritable bowel syndrome) that were impairing gastric emptying or colon motility, organic GI diseases (inflammatory bowel diseases, diverticular diseases, or motility disorders), or other organic diseases (neurological, endocrinal, or metabolic diseases) were excluded. Those with acute illnesses (upper respiratory tract infection, acute gastroenteritis) and those who were taking drugs (prokinetics, laxatives, stimulants, cholinergic, or anti-cholinergic drugs) that might influence the transit time were also excluded.

### Clinical and radiological assessment

The medical records included the initial location and progression of GI FBs as evident on serial radiographs, as well as clinical symptoms and signs. Round and spherical FBs were considered regularly shaped, and ovoid, long, flake-like, and projecting FBs were considered irregularly shaped. Peristalsis pushes irregularly shaped objects through the GI tract with the blunt end (e.g., the head of a screw) leading and the sharp end trailing (15, 16). The diameters of regularly shaped objects (coins, button batteries, etc.), and the diameters and lengths of irregularly shaped objects (screws, pins, magnets, etc.), were measured on radiographs.

### Correlation of FB size with transit time

Transit time was correlated with FB size. The cutoff diameters of regularly shaped objects, and the cutoff diameters and lengths of irregularly shaped objects, were subjected to statistical analysis. Mann-Whitney *U* test was performed to compare the means of continuous variables between independent samples. Comparisons between transit times of regularly shaped and irregularly shaped objects were made by Mann Whitney *U* test. Linear regression analysis was used to compare association between size (diameter or length) and transit time. A significance level of *p* < 0.05 was used.

Receiver operating characteristic (ROC) curve analysis was used to identify the different cutoff diameters or lengths for the prediction of slow transit time in FBs. An area under the curve (AUC) of 0.5 suggests no discrimination, 0.7–0.8 is considered acceptable, 0.8–0.9 is considered excellent, and >0.9 is considered outstanding (17). Cutoff values for the prediction of FBs with transit time >72 h were identified by ROC curve analysis.

### Ethics approval

The study was approved by the Institutional Review Board of Chang Gung Memorial Hospital, Linkou branch (approval no. 20160141B0).

## Results

**Figure 1:**
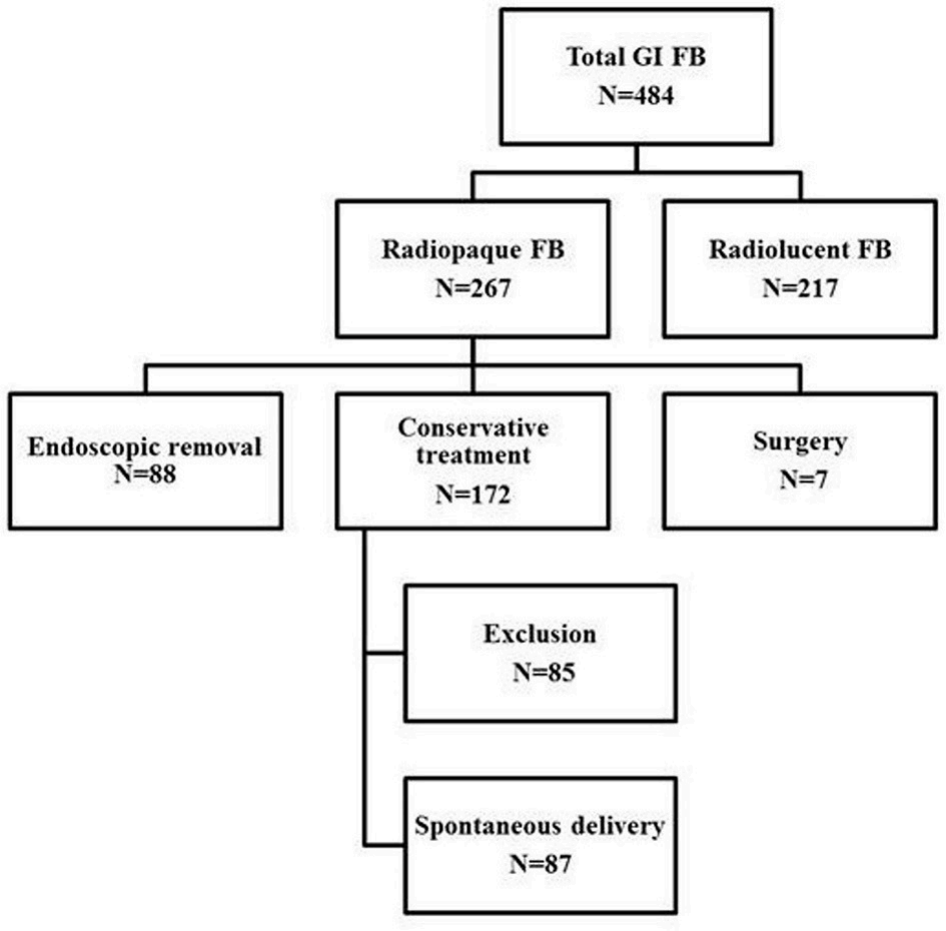
Algorithm for patient inclusion and classification.

In total, 484 patients with FBs in the GI tract were reviewed. Of the FBs, 267 (55.1%) were radiopaque. Of these 267 patients, 88 (33.0%) underwent endoscopic removal, 172 (64.4%) were clinically asymptomatic and so were monitored and subjected to serial radiography sessions, and 7 (2.6%) underwent surgery to treat perforations (*n* = 2) and bowel obstructions (*n* = 5). The algorithm for patient inclusion and classification is shown in Figure 1.

Of the 88 children who underwent endoscopic removal, 31.8% (28/88) were symptomatic (nausea, drooling, dysphagia, vomiting, or abdominal pain), 17% (15/88) had impacted FBs in the esophagus, 12.5% (11/88) had ingested button batteries, 8.0% (7/88) had ingested sharp objects, 6.8% (6/88) had ingested long objects (>5 cm), 20.4% (18/88) had swallowed large coins (diameter 22–30 mm), 3.4% (3/88) had ingested multiple magnets that were endoscopically accessible, and 2.2% (2/88) who initially refused endoscopic removal of coins (both 22 mm in diameter) returned to hospital for endoscopic removal after a 7-day observation period. Seven patients (five males and two females, aged 1–12 years) failed to spontaneously pass FBs and so underwent surgery. The clinical information of these seven patients is summarized in Table 1.

**Table 1:** Clinical data of the patients who underwent surgery due to failed delivery of gastrointestinal foreign bodies.

**Case**	**FB**	**Location**	**Condition**
1	Single magnet	Ileocecal valve	Stuck
2	Two magnets	Small intestine	Stuck
3	Needle	Ileocecal valve	Stuck
4	Wire	Ileocecal valve	Stuck and perforation
5	Iron line	Duodenal loop	Stuck
6	Pin	Duodenal loop	Stuck
7	Wire	Small intestine	Stuck and perforation

*FB, foreign body*.

**Figure 2:**
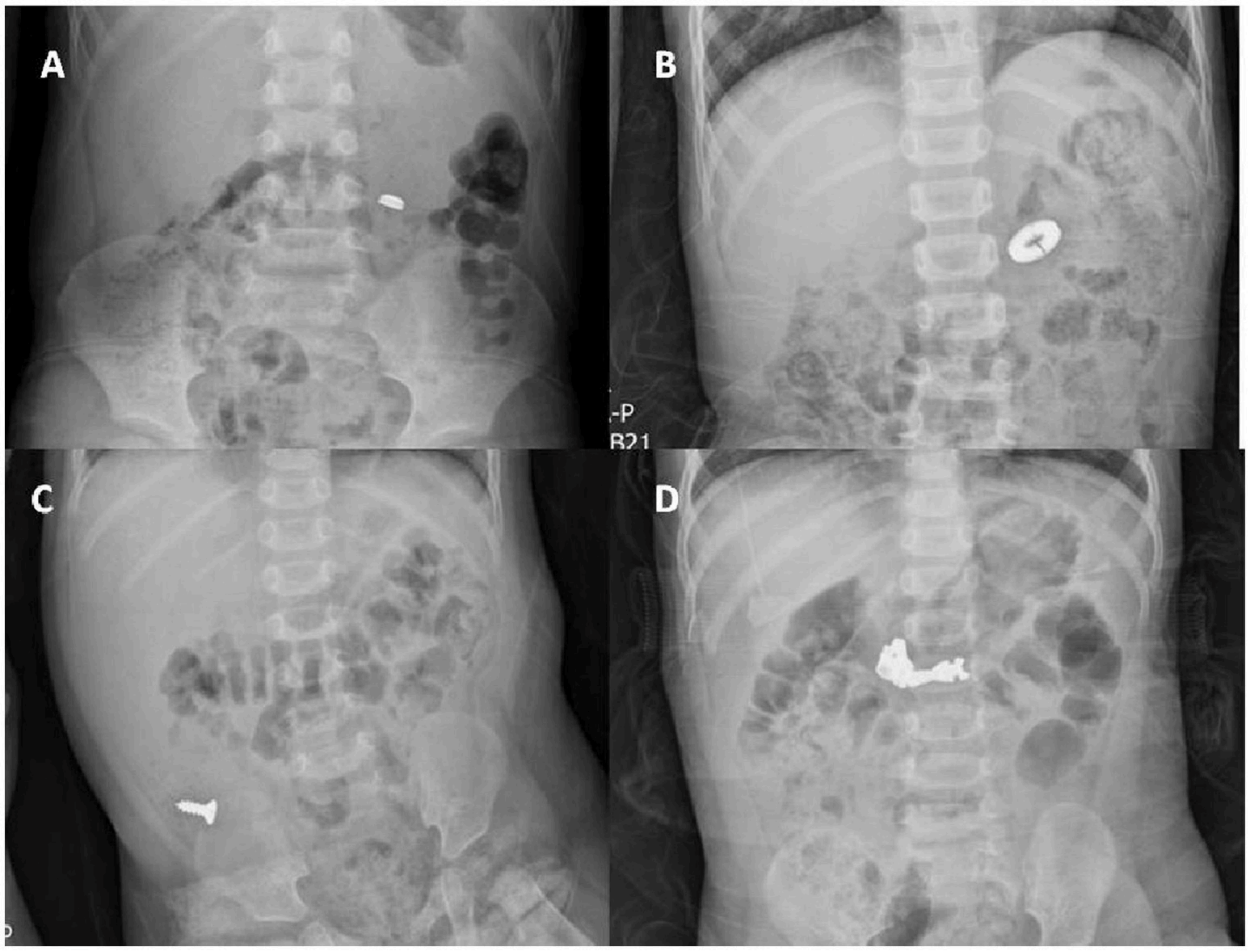
A plain X-ray of the abdomen showing regularly shaped FBs **(A,B)** and irregularly shaped FBs **(C,D)** in the GI tract. **(A)** A button disc battery in the small intestine. **(B)** A ring in the small intestine. **(C)** A screw in the colon. **(D)** A zipper slider in the small intestine.

All of the 172 patients who were monitored had radiographic confirmation of FB delivery at follow-up, while almost half (85/172 patients, 49.4%) were excluded because the precise transit times were lacking (*n* = 33), underlying diseases affected the transit time [neurological diseases (*n* = 12), chronic constipation (*n* = 17), acute illness (*n* = 23)]. A total of 87 patients for whom complete medical records were available (including gender, age, diameters of regularly shaped FBs, lengths of irregularly shaped FBs, and precise transit times) were finally enrolled. All 87 patients had ingested single FBs; 61 were regularly and 26 irregularly shaped. Coins were the most common FBs (*n* = 38, 43.6%), followed by button batteries or buttons (*n* = 17, 19.5%). The remaining FBs included screws, hair pins, zipper sliders, needles, keys, and metal toys. Figure 2 shows the various regularly and irregularly shaped FBs.

Basic data on these 87 patients are listed in Table 2. The male-to-female ratio was 2.0 (58 males, 29 females). Forty-nine (56.3%) patients were <3 years of age, and 73 (83.9%) were <5 years of age. The diameters of regularly shaped FBs ranged from 0.5 to 3.0 cm. The diameters and lengths of irregularly shaped FBs ranged from 0.2 to 0.9 cm and from 0.8 to 3.8 cm, respectively. The raw data of the size of regularly shaped and irregularly shaped FBs was shown in Supplementary Material (Data Sheet 1). The mean diameters of regularly shaped FBs were 1.63 ± 0.62 cm (mean ± SD), respectively. The mean diameters and lengths of irregularly shaped FBs were 0.45 ± 0.19 and 2.05 ± 0.70 cm, respectively. Compared with the irregularly shaped FBs, the regularly shaped FBs had longer transit times with greater variation, but the difference in transit time was not significant (*p* = 0.795 by Mann-Whitney *U* test).

**Table 2:** Demographic data and foreign body diameters in 87 patients with spontaneous delivery of gastrointestinal foreign bodies.

**FB categories**	**Regularly shaped**	**Irregularly shaped**
Number of patients	61	26
Age (mean ± SD)	3.62 ± 2.51 years	3.15 ± 2.68 years
Gender: male/female	40/21	18/8
Diameter^a^/length^b^ (mean *± SD)*	1.63 ± 0.62 cm^a^	2.05 ± 0.70 cm^b^
Transit time of spontaneous delivery	62.7 ± 95.7 h	51.2 ± 37.1 h
(mean ± SD, range)	(2.4–720)	(12–192)

*FB, foreign body; SD, standard deviation*.

**Figure 3:**
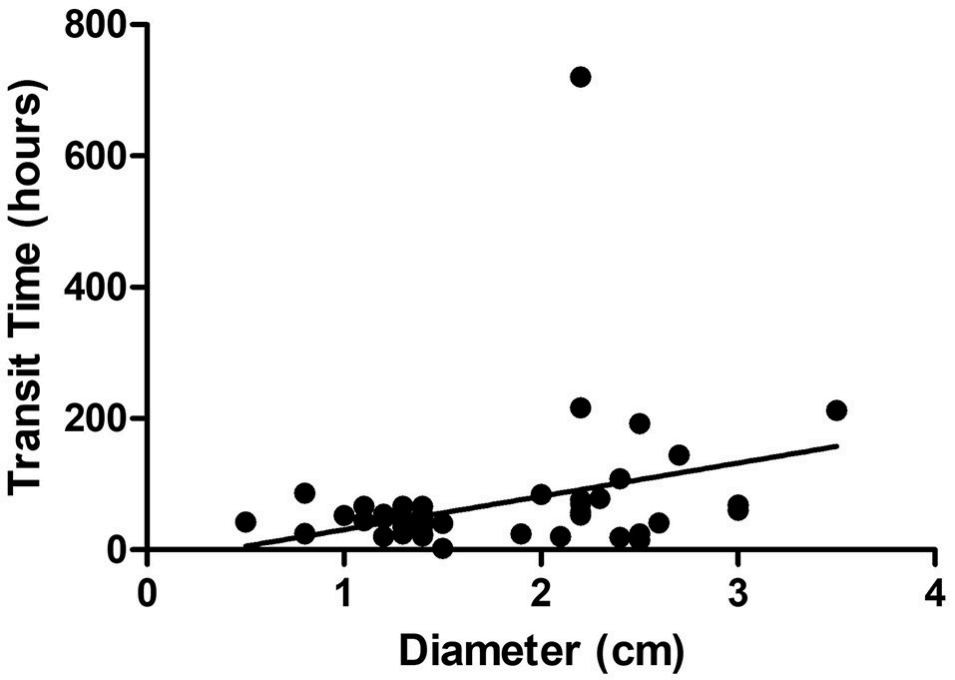
Linear regression analysis of the correlation between diameter and transit time in regularly foreign bodies.

**Figure 4:**
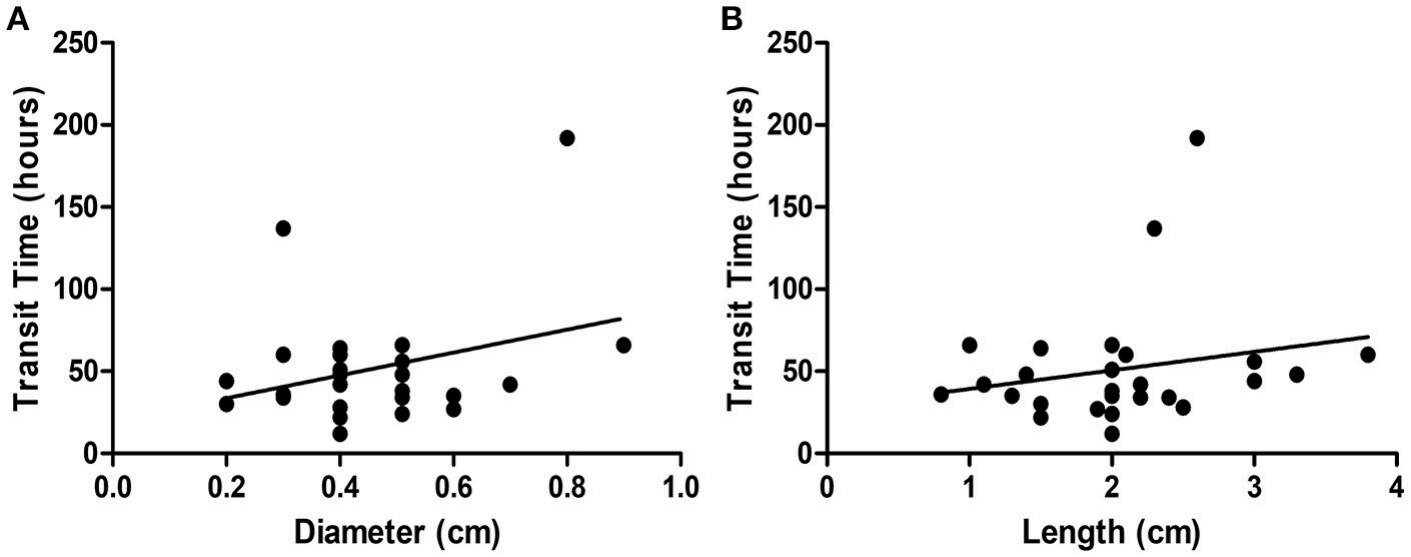
Linear regression analysis of the correlation between size [diameter **(A)**; length **(B)**] and transit time in irregularly foreign bodies.

**Figure 5:**
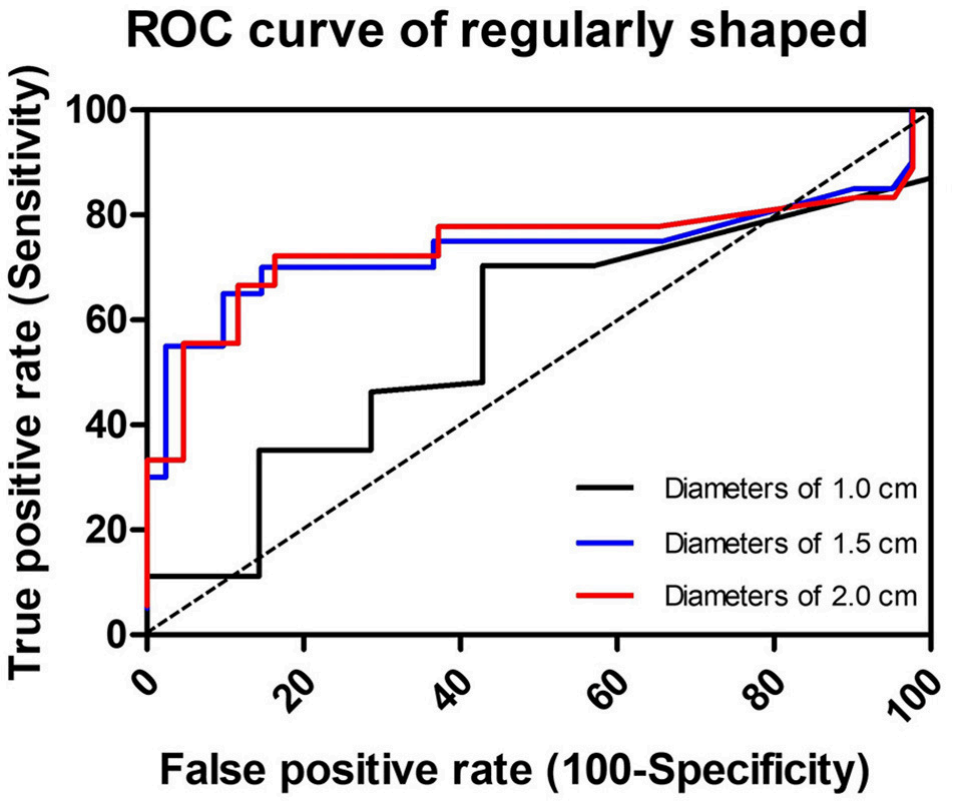
Receiver-operator characteristic curve (ROC) to illustrate the three cutoff diameters as predictors of slow transit in regularly shaped foreign bodies. The dotted line in the diagonal present the ROC curve of a random predictor (AUC: 0.5).

**Figure 6:**
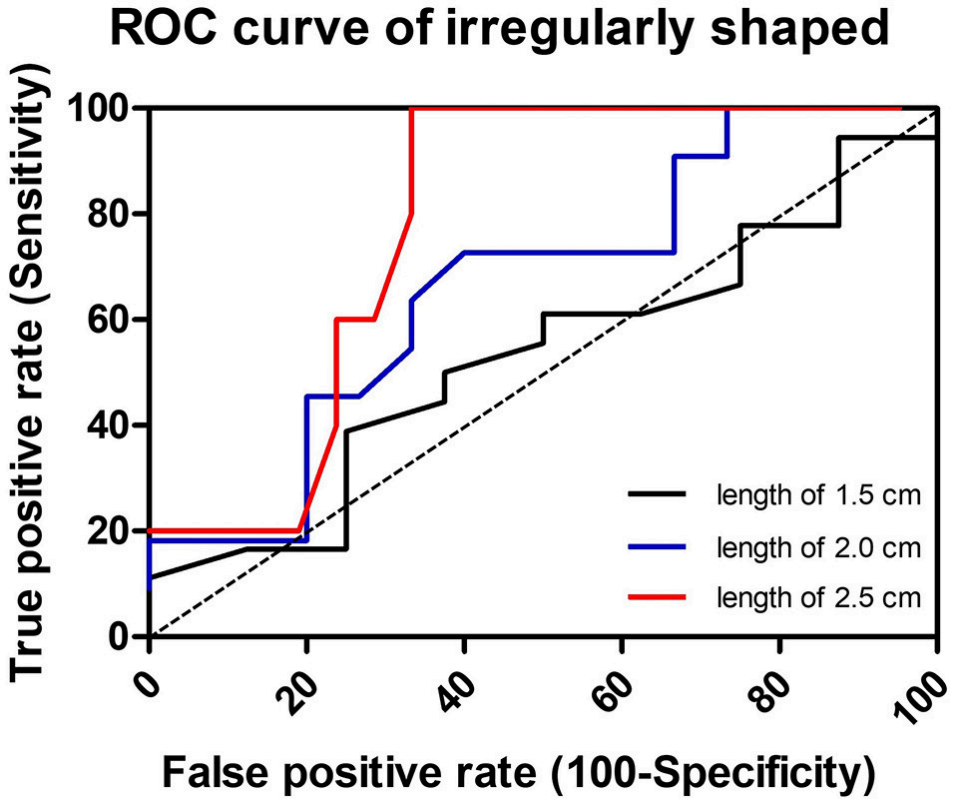
Receiver-operator characteristic (ROC) curve to illustrate the three cutoff lengths as predictors of slow transit in irregularly shaped foreign bodies. The dotted line in the diagonal presents the ROC curve of a random predictor (AUC: 0.5).

Three cutoff diameters (1.0, 1.5, and 2.0 cm) for regularly shaped FBs, and three cutoff lengths (1.5, 2.0, and 2.5 cm) for irregularly shaped FBs, were chosen for analysis of transit times (Tables 3, 4). Mann-Whitney *U* test showed that FBs 1.5 or 2.0 cm in diameter were associated with longer transit times than were FBs 1.0 cm in diameter. As shown in Table 3, diameter was positively correlated with transit time; a trend toward a longer transit time was observed as the diameter increased. At cutoffs of 1.5 and 2.0 cm, the *p*-values were both quite close to 0.003 (1.5 cm: 0.002966; 2.0 cm: 0.002969). Similarly, a trend toward a longer transit time was apparent as the length of irregularly shaped FBs increased, although no cutoff was statistically correlated with longer transit time. As shown in Table 4, the *p*-value for a cutoff diameter of 0.5 cm was 0.622; and the *p*-values for cutoff lengths of 1.5, 2.0, and 2.5 cm were 0.824, 0.153, and 0.055, respectively. Figures 3, 4 showed the linear regression lines between the size and transit time in regularly shaped and irregularly shaped objects, respectively. There were strong associations between diameter of regularly shaped objects and transit time (*p* = 0.0082, linear regression analysis), whereas no strong associations were observed in the irregularly shaped objects [*p* (diameter): 0.116; *p* (length): 0.2886]. ROC curve analysis showed that the optimal cutoff diameter to identify a longer transit time was 2 cm (AUC, 0.742, *p* = 0.003) for regularly shaped FBs, and the optimal cutoff length was 2.5 cm (AUC, 0.781, *p* = 0.055) for irregularly shaped FBs (Figures 5, 6 and Table 5). The optimal cutoff diameter for a transit time >72 h was 1.95 cm (AUC, 0.808, *p* = 0.002) for regularly shaped FBs, and the optimal cutoff length for a transit time >72 h was 2.25 cm (AUC, 0.792, *p* = 0.178) for irregularly shaped FBs (Table 6).

**Table 3:** Differences in the transit time of spontaneous delivery between different diameters of regularly shaped foreign bodies.

**FB**	**Diameter**		**Mann-Whitney**
	**≦1.0 cm**	**>1.0 cm**	*P*-value^*^
Regularly shaped (*n* = 61)	7	54	
Transit time (h) of spontaneous delivery [median (IQR)]	25 (24–47)	40.5 (24–64.5)	0.539
**FB**	**Diameter**		**Mann-Whitney**
	≦**1.5 cm**	>**1.5 cm**	***P***-value^*^
Regularly shaped (*n* = 61)	41	20	
Transit time (h) of spontaneous delivery [median (IQR)]	36 (24–48)	69 (36.75–117)	0.003^*^ (0.002966)
**FB**	**Diameter**		**Mann-Whitney**
	≦**2.0 cm**	>**2.0 cm**	***P***-value^*^
Regularly shaped (*n* = 61)	43	18	
Transit time (h) of spontaneous delivery [median (IQR)]	36 (24–48)	69 (44–135)	0.003^*^ (0.002969)

*FB, foreign body; IQR, interquartile range 25th to 75th percentiles*.

* *Data of median and IQR were compared by Mann-Whitney U test; data were significantly different between variables (p < 0.05)*.

**Table 4:** Differences in the transit time of spontaneous delivery between different diameters and lengths of irregularly shaped foreign bodies.

**FB**	**Diameter**		**Mann-Whitney**
	**≦0.5 cm**	**>0.5 cm**	*P*-value^*^
Irregularly shaped (*n* = 26)	15	11	
Transit time (h) of spontaneous delivery [median (IQR)]	42 (32–55.5)	42 (34.5–61)	0.622
**FB**	**Length**		**Mann-Whitney**
	≦**1.5 cm**	>**1.5 cm**	***P***-value^*^
Irregularly shaped (*n* = 26)	8	18	
Transit time (h) of spontaneous delivery [median (IQR)]	39 (33.75–52)	43 (34–59)	0.824
**FB**	**Length**		**Mann-Whitney**
	≦**2 cm**	>**2 cm**	***P***-value^*^
Irregularly shaped (*n* = 26)	15	11	
Transit time (h) of spontaneous delivery [median (IQR)]	36 (28.5–49.5)	48 (38–60)	0.153
**FB**	**Length**		**Mann-Whitney**
	≦**2.5 cm**	> **2.5 cm**	***P***-value^*^
Irregularly shaped (*n* = 26)	21	5	
Transit time (h) of spontaneous delivery [median (IQR)]	36 (30-51)	56 (48-60)	0.055

*FB, foreign body; IQR, interquartile range 25th to 75th percentiles*.

* *Data of median and IQR were compared by Mann-Whitney U test; data were significantly different between variables (p < 0.05)*.

**Table 5:** ROC curve analysis of different cutoff diameters and lengths for the prediction of slow transit time in foreign bodies.

	**Transit time (h)**	**AUC**	**Std. error**	***P*-value**	**95% CI**	
					**Lower bound**	**Upper bound**
**REGULARLY SHAPED FB**						
1 cm (diameter)	30	0.571	0.105	0.541	0.365	0.778
1.5 cm (diameter)	52.5	0.735	0.086	0.003	0.566	0.904
2 cm (diameter)	52.5	0.742	0.089	0.003	0.567	0.916
**IRREGULARLY SHAPED FB**						
1.5 cm (length)	43	0.528	0.122	0.824	0.290	0.766
2 cm (length)	43	0.667	0.108	0.154	0.455	0.879
2.5 cm (length)	43	0.781	0.092	0.055	0.601	0.961

*FB, foreign body; D, diameter; L, length; SD, standard deviation; AUC, area under the curve; Std., standard; CI, confidence interval. AUC: 0.5 (no discrimination), 0.7–0.8 (acceptable discrimination); 0.8–0.9 (excellent discrimination); 0.9–1.0 (outstanding discrimination)*.

**Table 6:** Receiver operating characteristics curve analysis of cutoffs for the prediction of foreign bodies with transit time >72 h.

**FB**	**Cutoffs for transit time > 72 h**	**AUC**	**Std. error**	***P*-value**	**95% CI**	
					**Lower bound**	**Upper bound**
Regularly shaped	1.95 cm (diameter)	0.808	0.088	0.002	0.636	0.979
Irregularly shaped	2.25 cm (length)	0.792	0.085	0.178	0.625	0.959

*FB, foreign body; SD, standard deviation; AUC, area under the curve; Std., standard; CI, confidence interval. AUC: 0.5 (no discrimination), 0.7–0.8 (acceptable discrimination); 0.8–0.9 (excellent discrimination); 0.9–1.0 (outstanding discrimination)*.

## Discussion

In this retrospective single center study we provide detailed information on transit time of the spontaneous GI passage of ingested FBs in pediatric patients and observed a close association in between the FB size and its transit time. We found that FB ingestion is more common in males than females; some studies have reported male-to-female ratios of approximately 1.5 (18); our ratio was 2.0. Similar to previous reports, we found that most FB ingestions occurred in children between the ages of 6 months and 5 years. Based on data of the American Association of Poison Control Centers' National Poison Data System (NPDS) (19), the peak age of FB ingestion is in the preschool years; >73% of FBs are ingested by children <5 years of age. Similarly, 83.9% of our patients were <5 years of age. Coins are commonly ingested and approximately two-thirds of all ingested FBs are reported to be adiopaque (20) compared to about 55% observed in our cohort. The characteristics of FBs ingested in our patients were similar to those of previous reports (coins, button batteries, and toys) (21–23).

Most FBs do not require immediate removal, as they are typically eliminated spontaneously (24–26). Previously published data show that approximately 10–20% of GI-tract FBs were removed endoscopically and 1% of patients required surgery (4, 27). In our study, 88/267 patients (33.0%) underwent endoscopic FB removal, and 7/267 (2.6%) required surgery because of perforation or bowel obstruction. our rates of endoscopic removal and surgical intervention were higher compared to previous reports (8, 13, 28, 29), perhaps because we enrolled only inpatients who, in the view of pediatric emergency room doctors, required endoscopic removal, or surgical intervention. Furthermore, our institution is the only tertiary care center in the three northern counties of Taiwan (population >5 million). Patients are referred to us from other hospitals or private clinics that lack endoscopic capacity.

The timing of endoscopy is dependent on several factors distinguishing in emergent and non-urgent cases and including the clinical status of the patient, the time of the patient's last oral intake, the type of ingested FB, and the location within the GI tract (30). The indications for an urgent endoscopic removal are the location of multiple magnets in the esophagus or stomach, button batteries in the esophagus, and sharp or long (>5 cm) objects in the esophagus or stomach (before the duodenal curve) (9, 30) such objects are more likely to induce severe esophageal trauma, triggering life-threatening complications requiring emergency surgery. FBs >2 cm in diameter or >3 cm in length in infants, and those 3–5 cm in length in children aged >1 year, may not pass through the GI tract and usually require endoscopic removal (31). When there is no movement of sharp or pointed objects (4 cm in length and 2 cm in diameter) for 3 days and of blunt objects for 7 days in the gastric or duodenal region, endoscopic or surgical exploration, and removal is recommended (32). Any FB that has not passed through the stomach within 3–4 weeks should be removed endoscopically (31, 33). Clinically, conservative treatment is usually suggested if endoscopic removal is not indicated. During observation, the family is usually worried. The recommended strategy is observation for 1–2 weeks; most FBs pass spontaneously in 4–6 days, although some require up to 4 weeks (34). The average transit time is 3.6 days regardless of FB size (21). The transit time in a series of pediatric patients observed by Hachimi-Idrissi et al. was 3.8 days (35). We provide transit time reference data in Tables 3, 4, indicating that the transit time was longer when the ingested objects were larger.

A few case reports have described complications arising during clinical observation. Coin retention in the cecum mimics appendicitis, and retention of a long object in the appendix, has in fact been known to cause appendicitis (36, 37). Furthermore, a severe complication (pyogenic liver abscess) caused by migration of a sharp object has been reported (38).

Surgery is primarily required in those with life-threatening complications such as bowel obstruction or perforation, trapped multiple magnets, lodged sharp objects, or objects trapped in regions of acute angulation, such as the ileocecal valve or rectal sigmoid colon (39). Seven of our patients required surgical intervention because of bowel obstruction, a lodged sharp object or perforation. Of these, three had impacted FBs at the ileocecal valve, two at the duodenal loop, and two in the small intestine. Our results support the suggestion that acute angulation or narrowing of the intestinal tract identifies areas at risk for FB impaction, and that wire FBs pose a risk of intestinal perforation.

The complication rate associated with sharp objects in the GI tract is 35% (40), although other case series have reported lower rates (41, 42). Sharp objects stuck in the esophagus require emergency care, and direct laryngoscopy is typically performed to remove objects lodged at or above the cricopharyngeus (13). Rigid or flexible endoscopy may be performed if laryngoscopy fails, or for handling of objects lodged below the cricopharyngeus. Sharp objects in the stomach or proximal duodenum should be retrieved endoscopically (13, 43, 44). In asymptomatic patients with sharp objects in the small intestine, serial radiography is recommended to evaluate the object's progress through the GI tract. Children with sharply pointed FBs are at high risk of intestinal tract impaction at locations that are acutely angled or narrow, such as the duodenal loop, the duodenojejunal junction, the appendix, and the ileocecal valve (41). Surgical intervention should be considered when objects fail to progress for 3 consecutive days, or if abdominal pain, vomiting, fever, hematemesis, or melena develop (30, 45). In our study, seven sharp objects were urgently retrieved from the stomach endoscopically, three (cases 3, 4, and 7; Table 1) of which were removed surgically; the others were located in the small intestine and passed spontaneously. The lengths of objects in the surgical cases varied from 3.8 to 4.5 cm, and were longer than the objects passed spontaneously (0.8–3.3 cm).

Strengths of our study include the use of strict exclusion criteria when enrolling subjects, close in-hospital observation of clinical symptoms, serial radiography, and precise records of the times of spontaneous FB passage. Although, our study has important practical implications for patients and clinicians, it has several limitations. First, this was a retrospective review, and it was therefore inherently biased in terms of patient selection. Although, we generally followed the guidelines for endoscopic FB removal, anxiety expressed by parents or caregivers sometimes caused us to perform aggressive endoscopic intervention. Second, the clinical protocols used to treat children with GI tract FBs were variable. Although, we excluded those who had undergone prior GI tract surgery and those with pre-existing GI tract abnormalities (congenital megacolon, strictures, diverticula, organic diseases, and functional GI disorders), fasting duration and meal types (which may influence transit time) were not controlled. The height and weight of the children were not analyzed in the present study. These may have been important variables, especially with respect to FB size. Thus, a further prospective study is warranted.

## Conclusion

The passage rate of ingested radiopaque FBs was 64.4%. Most small FBs pass spontaneously, but serious complications, such as bowel perforation and obstruction, can occur. FBs lodged in the esophagus should be removed endoscopically, and large or sharp FBs in the stomach or proximal duodenum must be removed as soon as possible. Patients who have ingested small smooth objects or objects that have passed the duodenal curve should be managed conservatively via clinical observation and radiographic surveillance. Our results indicate that the larger the size of a FB that has already passed the esophagus the longer its transit time will be; the transit time can vary from few hours to 30 days. When the diameter of a regularly shaped object is larger than 1.95 cm or the length of an irregularly shaped object is longer than 2.25 cm, the transit time is expected to be longer than 72 h.

## Ethics statement

This study was carried out in accordance with the recommendations of guidelines for Human Research, Chang Gung Memorial Hospital. The protocol was approved by the Institutional Review Board of the Human Research Committee of Chang Gung Memorial. All subjects gave written informed consent in accordance with the Declaration of Helsinki.

## Author contributions

H-CC contributed to the conception and design of the work. H-YY and H-CC drafted the manuscript. H-YY and S-YC conducted the analysis. C-CC and M-WL did the interpretation of the data. H-CC reviewed the final manuscript, and all the authors did the final approval of the version to be published.

### Conflict of interest statement

The authors declare that the research was conducted in the absence of any commercial or financial relationships that could be construed as a potential conflict of interest.

## Abbreviations

GI: gastrointestinal
FB: foreign body
SD: standard deviation.
